# Early versus late tracheostomy after decompressive craniectomy for stroke

**DOI:** 10.1186/s40560-017-0269-1

**Published:** 2018-01-04

**Authors:** Michael P. Catalino, Feng-Chang Lin, Nathan Davis, Keith Anderson, Casey Olm-Shipman, J. Dedrick Jordan

**Affiliations:** 1 Department of Neurosurgery, University of North Carolina School of Medicine, 170 Manning Drive, Campus Box 7025, Chapel Hill, NC 27599-7025 USA; 2 Department of Biostatistics, Gillings School of Global Public Health, Chapel Hill, NC USA; 3 School of Medicine, University of North Carolina School of Medicine, Chapel Hill, NC USA; 4 Department of Neurology, University of North Carolina, 170 Manning Drive, Campus Box 7025, Chapel Hill, NC USA

**Keywords:** Decompression, Ischemic stroke, Hemorrhagic stroke, Tracheostomy timing, Ventilator-associated pneumonia

## Abstract

**Background:**

Stroke patients requiring decompressive craniectomy are at high risk of prolonged mechanical ventilation and ventilator-associated pneumonia (VAP). Tracheostomy placement may reduce the duration of mechanical ventilation. Predicting which patients will require tracheostomy and the optimal timing of tracheostomy remains a clinical challenge. In this study, the authors compare key outcomes after early versus late tracheostomy and develop a useful pre-operative decision-making tool to predict post-operative tracheostomy dependence.

**Methods:**

We performed a retrospective analysis of prospectively collected registry data. We developed a propensity-weighted decision tree analysis to predict tracheostomy requirement using factors present prior to surgical decompression. In addition, outcomes include probability functions for intensive care unit length of stay, hospital length of stay, and mortality, based on data for early (≤ 10 days) versus late (> 10 days) tracheostomy.

**Results:**

There were 168 surgical decompressions performed on patients with acute ischemic or spontaneous hemorrhagic stroke between 2010 and 2015. Forty-eight patients (28.5%) required a tracheostomy, 35 (20.8%) developed VAP, and 126 (75%) survived hospitalization. Mean ICU and hospital length of stay were 15.1 and 25.8 days, respectively. Using GCS, SOFA score, and presence of hydrocephalus, our decision tree analysis had 63% sensitivity and 84% specificity for predicting tracheostomy requirement. The early group had fewer ventilator days (7.3 versus 15.2, *p* < 0.001) and shorter hospital length of stay (28.5 versus 44.4 days, *p* = 0.014). VAP rates and mortality were similar between the two groups. Withdrawal of treatment interventions shortly post-operatively confounded mortality outcomes.

**Conclusion:**

Early tracheostomy shortens duration of mechanical ventilation and length of stay after surgical decompression for stroke, but it did not impact mortality or VAP rates. A decision tree is a practical tool that may be helpful in guiding pre-operative decision-making with patients’ families.

**Electronic supplementary material:**

The online version of this article (10.1186/s40560-017-0269-1) contains supplementary material, which is available to authorized users.

## Background

Mechanically ventilated stroke patients are at risk for ventilator-associated pneumonia (VAP) and prolonged stay in intensive care units (ICU) [1–3]. Recent reviews cite the incidence of VAP to be 1–9 occurrences per 1000 ventilator days and suggest its pathogenesis to be multifactorial with timing, duration of endotracheal ventilation, host factors, and virulence of invading bacteria all contributing [4]. Patients with ischemic and hemorrhagic stroke can often wean rapidly from the ventilator after tracheostomy [5]. There is little evidence to guide timing of tracheostomy in patients with large hemispheric infarctions [6]. Generally, tracheostomy may be considered after 7–14 days if extubation is not feasible. Studies have looked at tracheostomy performed as early as hospital day 4 [7], but the definition of “early” and “late” varies widely in the literature [7–11]. Studies have shown a linear relationship between tracheostomy timing and ICU length of stay [3]. Furthermore, delayed tracheostomy may place patients at undue risk of pneumonia from prolonged mechanical ventilation [11–14].

The TRACH score [14] and SETscore [15] are two of the most comprehensive tools, among many that are used for predicting tracheostomy in patients with cerebrovascular injury [16]. The reported sensitivity of the TRACH score is 94% and has a specificity of 83% to predict extubation. Furthermore, the SETscore looks at neurological function, brain lesion factors, and general organ function to assess likelihood requiring greater than 2 weeks of ventilator support in stroke patients in the ICU [15]. A SETscore of 8 returned an optimum sensitivity of 65.4% and specificity of 73.5%. These are two helpful tools within a growing body of literature on this topic. In critically ill patients, studies are inconclusive but suggest a decrease in mortality and ICU length of stay and lower sedative requirement after early tracheostomy. [8, 17] The SETPOINT pilot study decreased ICU and 6-month mortality after tracheostomy placement 1–3 days after intubation in patients with all strokes, including subarachnoid hemorrhage, but indicated no change in ICU length of stay [9], although half of the patients randomized to standard tracheostomy died prior to receiving the intervention. We are critical in our interpretation of current literature as large well-controlled studies are still lacking, and those present are considerably heterogeneous. In one trial, about half of the patients assigned to late tracheostomy did not require the intervention at all [7]. Our objectives were to use our robust database and propensity weight methods to identify which factors predicted the need for tracheostomy in stroke patients requiring surgical decompression, as well as to analyze the relationship between the timing of tracheostomy, incidence of VAP, rate of in-hospital mortality, and ICU and hospital length of stay.

## Methods

### Data registry

The University of North Carolina (UNC) Neuroscience Intensive Care Unit (NSICU) patient registry is a prospectively collected database of all NSICU patients. Institutional Review Board approval was obtained to access the database for research purposes (IRB no. 15-2372). The database was queried. Inclusion criteria were as follows: adult patients, presentation between May 2010 and September 2015, ischemic or hemorrhagic stroke, and decompressive craniectomy. Data was extracted directly from the database, and patient records were reviewed for additional variables as needed. There were no exclusion criteria, and thus, all patients meeting the inclusion criteria were included in the initial analysis. Patients with missing variables were still included; however, specific missing variables excluded patients from the denominator of analyses where data was not available. In the final mortality analysis, patients who died on comfort care were excluded.

### Data analysis

Timing of tracheostomy was expressed in terms of post-stroke day or hospital day if the date of injury was indeterminate. A 10-day cutoff was preselected based on previous literature from a recent Cochrane Review [8]. It fell between the routine tracheostomy timing at our institution (7–14 days) and thus was also feasible to study using our database. The treatment group (early) included all patients receiving tracheostomy at or before stroke/hospital day 10, and the control group (late) was those who received a tracheostomy after day 10.

The primary objectives were to develop a predictive model for tracheostomy and compare outcomes for early versus late tracheostomy. Primary outcomes included mortality, ICU length of stay, hospital length of stay, and ventilator-associated pneumonia. Propensity weighting was used to predict the probability of tracheostomy after identifying crude predictors through bivariate analysis. We used propensity scores to control for differences in measured covariates between early and late tracheostomy cohorts by first estimating the probability of receiving tracheostomy based on crude bivariate analysis in Table 2. After weighting, this process leads to a pseudo-population, whose covariate distribution can be matched between early and late tracheostomy cohorts. This not only removes confounding by measured covariates, but also allows us to estimate the association between timing of tracheostomy and outcomes. We report percentages only in the final analysis because the actual counts are based on the pseudo-population. Variables considered for inclusion in the propensity weights were Glasgow Coma Score (GCS), time to surgery, hydrocephalus, location of stroke, and Sequential Organ Failure Assessment (SOFA) score. Time to surgery was not a clinically meaningful predictor after sensitivity analyses, and thus, it was not used in the final weights. GCS has been described as a predictor for tracheostomy after craniectomy for traumatic brain injury, but not for stroke, and thus, we felt it was a meaningful variable to include [18]. Hydrocephalus was defined as any neurological deterioration attributable to elevated ICP, which subsequently required cerebrospinal fluid diversion via a ventriculostomy drain. All primary outcomes were compared using propensity weights. Sensitivity analysis was performed for tracheostomy timing and primary outcomes.

## Results

**Fig. 1:**
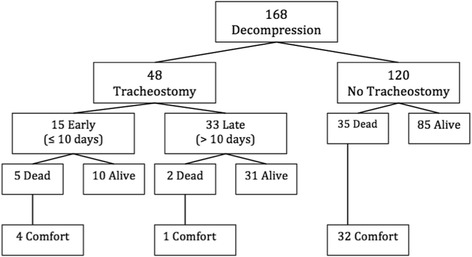
Flow chart showing the number of patients who underwent decompression for stroke (ischemic or hemorrhagic), those who received a tracheostomy, and survival, including those who were excluded from the final analysis due to death while on comfort-measures-only status

There were 168 patients who received surgical decompression following a stroke (Fig. 1). Descriptive patient characteristics are reported in Table 1. Average GCS on admission was 9.3, average time to surgery was 1.7 days, and average SOFA score was 6.5. There were 131 hemorrhagic and 37 ischemic strokes, of which 37 (28.2%) and 11 (29.7%), respectively, required a tracheostomy. Indications for tracheostomy included failure to wean mechanical ventilation for a prolonged period, neurological status that precludes extubation, and multiple failed extubation. Additionally, 35 (20.8%) patients developed VAP. On average, patients contracted VAP 2 days prior to receiving their tracheostomy and 9 days after admission (Table 1). Patients who required tracheostomy had longer ICU length of stay (27.8 versus 10.0 days, *p* < 0.001) and a longer duration of mechanical ventilation (13.0 versus 6.3 days, *p* < 0.001); however, these patients did not demonstrate a significantly longer mean length of hospital stay.

**Table 1:** Patient demographics and clinical factors by primary stroke etiology (hemorrhagic or ischemic)

	Total	ICH	Ischemic	*p* value
*N*	168	131 (78%)	37 (22%)	
Age, mean (SD)	55.3 (15.3)	55.3 (16.2)	55.2 (12.0)	0.116
Male	91 (54%)	65 (50%)	26 (70%)	0.039
Race				
White	86 (57%)	68 (59%)	18 (53%)	0.547
African American	51 (34%)	37 (32%)	14 (41%)	
Other	13 (9%)	11 (9%)	2 (6%)	
BMI, mean (SD)	28.1 (7.4)	27.6 (7.6)	29.7 (6.2)	0.148
Myocardial infarction	8 (5%)	6 (5%)	2 (5%)	1.000
Congestive heart failure	9 (5%)	3 (2%)	6 (16%)	0.004
Peripheral vascular disease	11 (7%)	6 (5%)	5 (13%)	0.067
Dementia	1 (1%)	1 (1%)	0 (0%)	1.000
Cerebrovascular disease	23 (14%)	19 (15%)	4 (11%)	0.787
Chronic lung disease	17 (10%)	14 (11%)	3 (8%)	0.766
Ulcer	3 (2%)	3 (2%)	0 (0%)	1.000
Chronic liver disease	6 (4%)	6 (5%)	0 (0%)	0.340
Diabetes	30 (18%)	18 (14%)	12 (32%)	0.015
Moderate–severe kidney disease	13 (8%)	13 (10%)	0 (0%)	0.074
Diabetes with organ damage	4 (2%)	4 (3%)	0 (0%)	0.577
Tumor	10 (6%)	9 (7%)	1 (3%)	0.461
Leukemia	3 (2%)	3 (2%)	0 (0%)	1.000
Lymphoma	0 (0%)	0 (0%)	0 (0%)	NA
Moderate–severe liver disease	1 (1%)	1 (1%)	0 (0%)	1.000
Malignant tumor	9 (5%)	8 (6%)	1 (3%)	0.685
Metastasis	5 (3%)	5 (4%)	0 (0%)	0.588
AIDS	3 (2%)	1 (1%)	2 (5%)	0.124
Hosp. LOS, mean (SD)	25.8 (27.0)	27.1 (29.2)	21.2 (16.2)	0.246
Discharge location				
SNF	35 (27.8%)	26 (26.8%)	9 (31.0%)	0.560
AIR	54 (42.9%)	40 (41.2%)	14 (48.3%)	
Home	25 (19.8%)	22 (22.7%)	3 (10.3%)	
LTAC	10 (7.9%)	7 (7.2%)	3 (10.3%)	
Hospice	2 (1.6%)	2 (2.1%)	0 (0.0%)	
Discharge condition				
Alive	126 (75.0%)	97 (74.0%)	29 (78.4%)	0.671
Dead without comfort care	5 (3.0%)	4 (3.1%)	1 (2.7%)	
Dead with comfort care	37 (22.0%)	30 (22.9%)	7 (18.9%)	
ICU LOS, mean (SD)	15.1 (16.2)	16.0 (17.7)	11.9 (8.8)	0.190
Readmission to ICU	37 (22.2%)	26 (20.0%)	11 (29.7%)	0.261
GCS on adm., mean (SD)	9.3 (3.7)	8.9 (3.8)	10.6 (3.2)	0.012
Admission mRS, mean (SD)	4.9 (0.4)	4.9 (0.5)	5.0 (0.0)	0.068
SOFA score, mean (SD)	6.5 (2.8)	6.6 (2.8)	6.1 (2.7)	0.331
NIHSS, mean (SD)	18.3 (7.2)	–	18.3 (7.2)	NA
Location				
Bilateral supratentorial	8 (4.8%)	8 (6.1%)	0 (0.0%)	0.456
Infratentorial	32 (19.0%)	24 (18.3%)	8 (21.6%)	
Left supratentorial	57 (33.9%)	45 (34.4%)	12 (32.4%)	
Right supratentorial	71 (42.3%)	54 (41.2%)	17 (45.9%)	
ICH score, mean (SD)	1.81 (0.95)	1.81 (0.95)	–	NA
IVH	62 (48.1%)	62 (48.1%)	–	NA
Hydrocephalus	88 (68.8%)	88 (68.8%)	–	
Time to surgery, mean (SD)	1.70 (3.6)	1.70 (3.9)	1.68 (2.4)	0.969
Time to ventilator, mean (SD)	0.65 (2.4)	0.63 (2.7)	0.73 (1.3)	0.821
Duration of ventilator, mean (SD)	8.1 (7.7)	8.8 (8.1)	5.9 (5.4)	0.051
# failed weans, mean (SD)	0.37 (0.56)	0.36 (0.57)	0.38 (0.55)	0.873
Tracheostomy	48 (28.6%)	37 (28.2%)	11 (29.7%)	0.840
Hosp tracheostomy day, mean (SD)	13.8 (7.2)	14.4 (7.9)	11.8 (4.0)	0.301
Duration tracheostomy, mean (SD)	29.8 (28.3)	30.2 (27.2)	28.3 (33.3)	0.912
Tracheostomy at discharge	39 (81.3%)	28 (75.7%)	11 (100%)	0.070
Total TRACH score	2.3 (2.4)	2.3 (2.4)	–	NA
Number of VAP, mean (SD)	0.56 (1.22)	0.59 (1.27)	0.46 (1.04)	0.574
Hospital VAP day, mean (SD)	9.5 (11.2)	9.2 (11.6)	10.3 (10.6)	0.826
Tracheostomy VAP day, mean (SD)	− 2 (16.6)	− 4.2 (17.8)	3.2 (13.4)	0.421

*AIR* acute inpatient rehabilitation, *GCS* Glasgow Coma Score, *ICU* intensive care unit, *LOS* length of stay, *LTAC* long-term acute care, *SD* standard deviation, *SNF* skilled nursing facility, *SOFA* Sequential Organ Failure Assessment

**Fig. 2:**
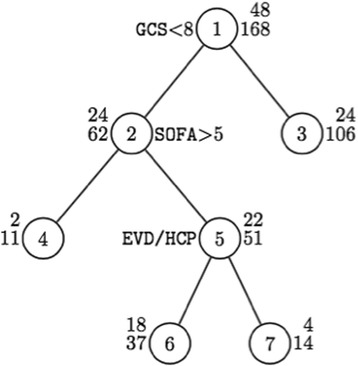
Decision tree for the prediction of tracheostomy. At each split, a patient goes to the left branch when the left-side condition is satisfied and goes to the right branch when the right-side condition is satisfied. The top number (numerator) is the number of patients who received a tracheostomy. The bottom number (denominator) is the sample size for that group. Assessing 2 and 3 results in odds ratio 2.14 with 95% CI 1.03–4.52 and *p* value = 0.034, 50% sensitivity, and 68% specificity. Assessing 4 and 5 results in odds ratio 3.35 with 95% CI 0.61–35.0 and *p* value = 0.178; sensitivity improves to 54% and specificity to 76%. Finally, assessing 6 and 7 results in odds ratio 2.33 with 95% CI 0.54–12.1 and *p* value = 0.225; combining three classification criteria, the sensitivity is 63% and the specificity is 84%

Bivariate analysis comparing covariates among those who received a tracheostomy and those who did not are shown in Table 2. These covariates were used to build propensity weights as shown in Tables 3 and 4. Duration of mechanical ventilation was the only significant variable that correlated with VAP in multivariate regression modeling. Lower GCS, higher SOFA, and hydrocephalus all were associated with higher likelihood of receiving tracheostomy (Tables 3 and 4), and these variables were included in the decision tree analysis (Fig. 2).

**Table 2:** Bivariate analysis for receiving a tracheostomy

	No trach	Trach	*p* value
*N*	120	48	
Diagnosis			
ICH	94 (78.3%)	37 (77.1%)	0.860
Ischemic	26 (21.7%)	11 (22.9%)	
Age, mean (SD)	56.4 (14.6)	52.4 (16.9)	0.129
Male	65 (54%)	26 (54%)	1.000
Race			
White	61 (58%)	25 (56%)	0.740
African American	34 (32%)	17 (38%)	
Other	10 (10%)	3 (7%)	
BMI, mean (SD)	27.5 (5.9)	29.4 (10.1)	0.133
GCS on adm., mean (SD)	9.7 (3.7)	8.2 (3.6)	0.023
Adm. mRS, mean (SD)	4.92 (0.50)	5.0 (0)	0.246
SOFA score, mean (SD)	6.2 (2.7)	7.1 (2.9)	0.055
NIHSS, mean (SD)	18.1 (6.8)	18.7 (8.7)	0.853
Location			
Bilateral supratentorial	5 (4.2%)	3 (6.3%)	0.638
Infratentorial	23 (19.2%)	9 (18.8%)	
Left supratentorial	38 (31.7%)	19 (39.6%)	
Right supratentorial	54 (45.0%)	17 (35.4%)	
ICH score, mean (SD)	1.77 (1.00)	1.91 (0.82)	0.458
IVH	41 (44.6%)	21 (56.8%)	0.210
Hydrocephalus	58 (63.7%)	30 (81.1%)	0.055
Time to surgery, mean (SD)	1.23 (2.19)	2.85 (5.77)	0.009
VAP	17 (14.2%)	18 (37.5%)	0.001
Number of VAP, mean (SD)	0.38 (1.00)	1.02 (1.56)	0.002

**Table 3:** Propensity-weighted outcomes for timing of tracheostomy

	Early	Late	*p* value
Mortality	33.3%	6.1%	0.006
VAP	40.0%	36.4%	0.614
Duration of ventilation, mean days (SD)	7.3 (7.2)	15.2 (6.6)	< 0.001
ICU stay, mean days (SD)	20.1 (10.6)	31.5 (28.1)	0.073
Hospital stay, mean days (SD)	28.5 (12.5)	44.4 (33.7)	0.014
Discharge location			
Home/rehabilitation	40.0%	29.0%	0.192
Skilled nursing facility/LTAC	60.0%	71.0%

**Table 4:** Propensity-weighted outcomes for timing of tracheostomy (excluding those who died on comfort care)

	Early	Late	*p* value
Mortality	9.1%	3.1%	0.780
VAP	36.4%	37.5%	0.652
Duration of ventilation, mean days (SD)	5.5 (3.4)	15.2 (6.7)	< 0.001
ICU stay, mean days (SD)	20.2 (10.2)	32.0 (28.4)	0.153
Hospital stay, mean days (SD)	31.3 (11.7)	45.3 (33.8)	0.075
Discharge location			
Home/rehabilitation	40.0%	29.0%	0.192
Skilled nursing facility/LTAC	60.0%	71.0%

### Decision tree analysis

GCS was the most important predictor for tracheostomy. Patients with a GCS less than 8, SOFA greater than 5, and hydrocephalus had the highest likelihood of requiring tracheostomy. Combining these classification criteria, the sensitivity is 63% and the specificity is 84%. Figure 2 shows the decision tree analysis, and the caption provides a detailed explanation of its interpretation.

### Primary outcomes and tracheostomy timing

Fifteen patients (31.2%) received a tracheostomy prior to hospital day 10, and 33 patients received a tracheostomy after day 10 (68.8%). Propensity-weighted outcomes comparing no tracheostomy versus tracheostomy were reviewed. Patients who received a tracheostomy had comparable mortality (after excluding those who transitioned to comfort measures), higher VAP incidence, and longer ICU length of stay (Additional file 1: Table S2). Early tracheostomy significantly predicted mortality compared to late tracheostomy [10/15 (33.3%) versus 2/33 (6.1%), respectively, *p* = 0.006; Table 3] although this was completely explained by early withdrawal of treatment and death while on comfort care (Table 4). Early tracheostomy did not protect against VAP compared to late tracheostomy (40.0% versus 36.4%, *p* = 0.614). Early tracheostomy did lead to less overall duration of ventilator dependence. Mean ventilator days for early tracheostomy was 7.3 versus 15.2 days for late tracheostomy (*p* < 0.001). Early tracheostomy was associated with a trend toward reducing the ICU length of stay, 20.1 versus 31.5 days, although this difference was not significantly different (*p* = 0.073; Table 3). Early tracheostomy significantly reduced hospital length of stay from 44.4 to 28.5 days (*p* = 0.014; Table 3).

**Fig. 3:**
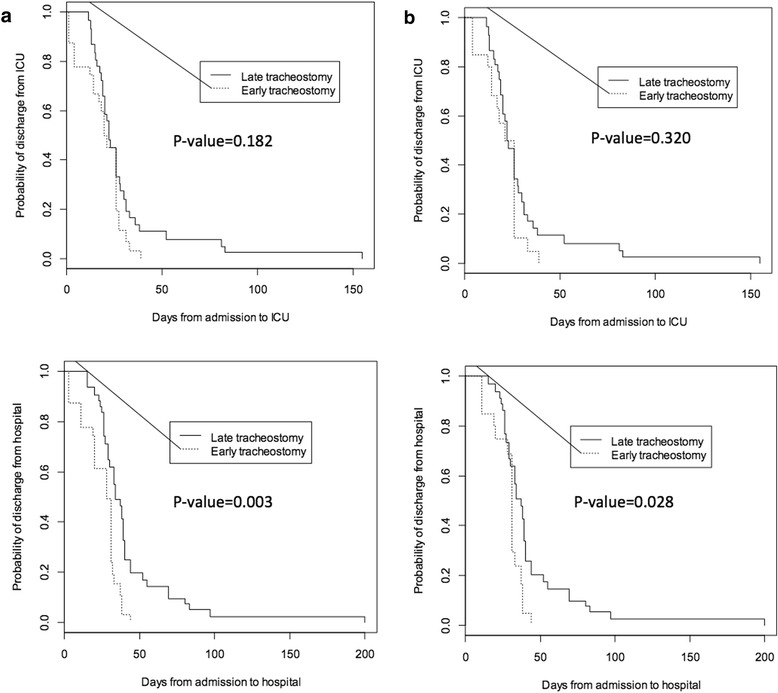
**a** Propensity-weighted probability functions for time-to-event for discharge from ICU (top) and discharge from the hospital (bottom) based on timing of tracheostomy. **b** Propensity-weighted probability functions for time-to-event for discharge from ICU (top) and discharge from the hospital (bottom) based on timing of tracheostomy (excluding those who died on comfort care)

**Fig. 4:**
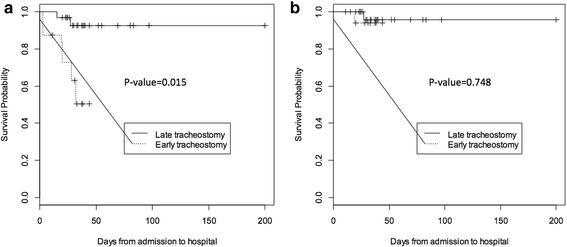
**a** Propensity-weighted Kaplan-Meier survival curve. **b** Propensity-weighted Kaplan-Meier survival curve (excluding those who died on comfort care)

VAP incidence rate was similar for both groups, 40% for early versus 36% for late tracheostomy (*p* = 0.614). If the cutoff is changed to day 7 or earlier, the VAP is much lower for the early group (20 versus 40%, *p* = 0.821), but statistical significance is not reached due to too few patients in this group. For discharge location, early tracheostomy showed a trend toward more favorable discharge locations (40.0 versus 29.0%, *p* = 0.192; Table 4). According to propensity-weighted probability functions for hospital discharge, the early tracheostomy group had a significantly shorter hospital length of stay (Fig. 3). However, propensity-adjusted mortality rate analysis resulted in a significantly higher mortality rate in patients who received an early tracheostomy (33.3 versus 6.1%, *p* = 0.006; Table 3 and Fig. 4a). In addition, patients with early tracheostomies still trended toward favorable discharge (home or to rehab) compared to those with late tracheostomies (40.0% versus 29.0%, respectively) with similar VAP rates (36.4% versus 37.5%, respectively; Table 4).

## Discussion

Predicting who will require a tracheostomy after decompressive surgery for ischemic stroke or intracerebral hemorrhage remains a challenge. Furthermore, we, and others, have shown that the risk of ventilator-associated pneumonia is strongly associated with duration of mechanical ventilation [2]. Predicting which of these critically brain-injured patients will ultimately need a tracheostomy would be helpful when discussing treatment options with families. We have developed a decision tree, based on key variables present on admission (GCS, SOFA score, and presence of hydrocephalus; Fig. 2), which can help guide clinical judgment as to who may be a candidate for tracheostomy. This adds to the repertoire of clinical decision-making tools available to help expedite tracheostomy placement and free patients from the ventilator. Here, we argue that early tracheostomy may expedite ICU and hospital discharge, but its impact on reducing the risk of ventilator-associated pneumonia and mortality is still unclear.

In the initial TRACH score study, all patients with TRACH score > 2.0 required a tracheostomy, while none with a score < 0.7 required a tracheostomy. In our data, ICH patients requiring decompressive surgery and no tracheostomy had a mean TRACH score of 2.2 and 3.2 for those who did have a tracheostomy (*p* = 0.041). Thus, if using the TRACH score cutoff of 2, as in the original study, many of our patients would have received a tracheostomy that did not ultimately need one [14].

Our decision analysis shows that, in patients with a GCS less than 8, SOFA greater than 5, and hydrocephalus requiring a ventriculostomy, the sensitivity is 63% and the specificity is 84% for predicting tracheostomy requirement. This results in a positive predictive value of 61.2% and a negative predictive value of 85%. Our primary outcome data is similar to the results of a recent meta-analysis by McCredie and colleagues looking at early tracheostomy in patients with severe acute brain injury [19]. They found that although early tracheostomy reduced length of ICU stay, it did not have a significant mortality benefit. These results are in contrast to another study from Brazil which showed a drastically reduced 28-day mortality rate in early tracheostomy patients (9 versus 46%, *p* = 0.049) even with a small sample size (*n* = 28) [20]. Notably, GCS and SOFA score were similar in the two groups, serving as an internal control. However, they also noted an extremely high rate of VAP in the late group (54% in the early group and 70% in the late group), whereas our VAP rate was 40.0% in the early group and 36.4% in the late group. This may help explain the mortality benefit in their population, since higher VAP rates combined with rapid weaning after tracheostomy will reduce total exposure to risk of VAP. Even though the VAP rates were not significantly different, as was true in our study, we suspect this was due to inadequate study power. Despite lack of direct evidence, we still believe early tracheostomy likely reduces total ventilator time eliminating the primary risk factor for VAP, the ventilator itself. Given the reduction in ICU length of stay, there are likely economic benefits from early tracheostomy as well. This was not explicitly studied here. Ongoing prospective controlled trials are needed to ultimately provide sound evidence for true early tracheostomy in these patients.

The trend in mortality was unexpected, so we reviewed all deaths after our final analysis to try and understand this early mortality phenomenon. We found that a large number of patients died after the family decided to withdraw treatment and transition to comfort care. A total of 42 patients died after decompression, and 37 (88.1%) of them died after transitioning to comfort measures. Among the five others, only two had tracheostomies making the sample too small to draw conclusions on timing of tracheostomy and mortality (Tables 3 and 4 and Fig. 4). However, even after excluding patients who died on comfort care, duration of mechanical ventilation and ICU and hospital length of stay were still significantly shorter in patients with early tracheostomies (Table 4 and Fig. 3b). In conclusion, our propensity-adjusted dataset shows that those who received an early tracheostomy actually trended toward a higher mortality rate (Table 3; Fig. 4a), but this was completely explained by early transition to comfort measures.

The basis of transition to comfort measures is usually a failure to see measurable improvement combined with a poor expected prognosis. This can materialize in various forms in patients with severe brain injuries. It may result in a “prognostic pessimism” [21] or “self-fulfilling prophecy” [22] in patients who are young and have a reasonable chance of survival. In older patients, where withdrawal of support is unlikely to change the outcome, it may be a reasonable decision based on prolonged suffering without clear long-term benefit. The decision to undergo emergent neurosurgery can be even more challenging, as there is often inadequate time and data to make a decision of such importance. Furthermore, prognostic models ideally would have perfect discrimination, with a 0% false positive rate for poor outcome [21]. However, this is currently only available for patients with anoxic brain injury after cardiopulmonary resuscitation, and oftentimes, families just need time under aggressive treatment to cope with the finality of the patient’s condition [21].

Our mean patient age was 55 years old, which puts the majority of our patients within the data-supported range for mortality benefit from decompression for ischemic stroke [23–25]. For intracranial hemorrhage, the benefit of surgical decompression is less clear and is often a last resort in the face of cerebral herniation and brainstem compression. When asked about the maximum age compatible with meaningful survival in patients with intracerebral hemorrhage, responders quoted about 70 years [4]. These same authors found that medical support was ultimately withdrawn in about 76.7% of patients who died, which is close to our finding of 88.1%. The decision of medical futility will ultimately fall on the surgeon and intensivist providing the treatment. Predicting tracheostomy dependence may help families with the surgical decision as well, although, even with our decision tree analysis, models remain imperfect. Sometimes, even after maximal intervention and attempts sustain the life of stroke patients, the lack of measureable improvement and small setbacks breach the threshold families have for continuing with the treatment they once desired for their loved one.

## Conclusions

The natural history of acute ischemic and hemorrhagic stroke requiring surgical decompression carries a grim prognosis. Early tracheostomy seems to have a few measurable benefits, namely shorter duration of mechanical ventilation and shorter length of stay. These may, however, be significant enough to families, and early tracheostomy may be reasonable to consider. We developed a decision tree analysis that can be used by neurosurgeons and neurointensivists to aid families in their decision-making prior to surgery. Upon ICU admission, surrogate decision-makers often have little time to process these tragic circumstances, and committing someone to a major operation, even if life saving, is often a difficult choice to make. An artificial airway is a concrete outcome laypersons can understand. We believe that these types of tools are useful to inform surrogates, guide appropriate care, and limit unnecessarily aggressive interventions leading to early post-operative withdrawal of treatment and early death.

## Additional file

Additional file 1: Table S1. Bivariate analysis for ventilator associated pneumonia. **Table S2.** Outcome analysis with and without tracheostomy using proposed propensity score. (DOCX 15 kb)

## Abbreviations

GCS: Glasgow Coma Score
ICU: Intensive care unit
SOFA: Sequential Organ Failure Assessment
VAP: Ventilator-associated pneumonia
