# Supplementary material for: Early versus late tracheostomy after decompressive craniectomy for stroke
Source: J Intensive Care. 2018 Jan 4;6:1. doi: 10.1186/s40560-017-0269-1 (PMC5753520; doi:10.1186/s40560-017-0269-1)
Supplement: Additional file 1: Table S1. — Bivariate analysis for ventilator associated pneumonia. Table S2. Outcome analysis with and without tracheostomy using proposed propensity score. (DOCX 15 kb) [file 40560_2017_269_MOESM1_ESM.docx]

Additional file 1: Table S1 Bivariate analysis for ventilator associated pneumonia

|  | **Total** | **No VAP** | **VAP** | **p-value** |
| --- | --- | --- | --- | --- |
| N | 168 | 133 | 35 |  |
| Diagnosis |  |  |  |  |
| ICH | 131 (78.0%) | 104(78.2%) | 27 (77.1%) | 1.000 |
| Ischemic | 37 (22.0%) | 29 (21.8%) | 8 (22.9%) |  |
| Age, mean (SD) | 55.3 (15.3) | 55.8 (15.5) | 53.2 (14.8) | 0.368 |
| Male | 91 (54%) | 69 (52%) | 22 (63%) | 0.260 |
| Race |  |  |  |  |
| White | 86 (57%) | 68 (58%) | 18 (56%) | 0.983 |
| African American | 51 (34%) | 40 (34%) | 11 (34%) |  |
| Other | 13 (9%) | 10 (8%) | 3 (10%) |  |
| BMI, mean (SD) | 28.1 (7.4) | 28.3 (7.8) | 27.2 (5.7) | 0.437 |
| Hospital LOS, mean (SD) | 25.8 (27.0) | 22.7 (21.9) | 37.3 (39.2) | 0.004* |
| Discharge Location |  |  |  |  |
| SNF | 35 (27.8%) | 26 (25.7%) | 9 (36.0%) | 0.136 |
| AIR | 54 (42.9%) | 45 (44.6%) | 9 (36.0%) |  |
| Home | 25 (19.8%) | 23 (22.8%) | 2 (8.0%) |  |
| LTAC | 10 (7.9%) | 6 (5.9%) | 4 (16.0%) |  |
| Hospice | 2 (1.6%) | 1 (1.0%) | 1 (4.0%) |  |
| Discharge Condition |  |  |  |  |
| Alive | 126 (75.0%) | 101 (75.9%) | 25 (71.4%) | 0.548 |
| Dead wo Comfort Care | 5 (3.0%) | 3 (2.3%) | 2 (5.7%) |  |
| Dead w Comfort Care | 37 (22.0%) | 29 (21.8%) | 8 (22.9%) |  |
| ICU LOS, mean (SD) | 15.1 (16.2) | 12.1 (10.0) | 27.0 (27.3) | 0.004* |
| Readmission to ICU | 37 (22.2%) | 26 (19.7%) | 11 (31.4%) | 0.169 |
| GCS on adm., mean (SD) | 9.3 (3.7) | 9.3 (3.8) | 9.1 (3.5) | 0.773 |
| Adm. mRS, mean (SD) | 4.9 (0.4) | 4.95 (0.32) | 4.89 (0.68) | 0.387 |
| SOFA score, mean (SD) | 6.5 (2.8) | 6.5 (2.8) | 6.3 (2.8) | 0.757 |
| NIHSS, mean (SD) | 18.3 (7.2) | 17.5 (8.0) | 20.8 (2.6) | 0.123 |
| Location |  |  |  |  |
| Bilateral supratentorial | 8 (4.8%) | 7 (5.3%) | 1 (2.9%) | 0.774 |
| Infratentorial | 32 (19.0%) | 25 (18.8%) | 7 (20.0%) |  |
| Left supratentorial | 57 (33.9%) | 43 (32.3%) | 14 (40.0%) |  |
| Right supratentorial | 71 (42.3%) | 58 (43.6%) | 13 (37.1%) |  |
| ICH score, mean (SD) | 1.81 (0.95) | 1.77 (0.97) | 1.96 (0.87) | 0.371 |
| IVH | 62 (48.1%) | 45 (44.1%) | 17 (63.0%) | 0.089 |
| Hydrocephalus | 88 (68.8%) | 64 (62.7%) | 24 (92.3%) | 0.004* |
| Time to surgery, mean (SD) | 1.7 (3.65) | 1.52 (3.44) | 2.37 (4.35) | 0.220 |
| Time to ventilator, mean (SD) | 0.65 (2.4) | 0.72 (2.69) | 0.37 (1.11) | 0.453 |
| Duration of vent., mean (SD) | 8.1 (7.7) | 7.4 (7.9) | 11.2 (6.1) | 0.011* |
| # failed weans, mean (SD) | 0.37 (0.56) | 0.31 (0.53) | 0.59 (0.66) | 0.009* |
| Tracheostomy | 48 (28.6%) | 30 (22.6%) | 18 (51.4%) | 0.001* |
| Hosp. trach. day, mean (SD) | 13.8 (7.2) | 14.0 (8.1) | 13.6 (5.6) | 0.851 |
| Duration of trach., mean (SD) | 29.8 (28.3) | 26.3 (23.8) | 35.5 (34.5) | 0.996 |
| Trach. at Discharge | 39 (81.3%) | 25 (83.3%) | 14 (77.8%) | 0.711 |
| Total Trach Score | 2.3 (2.4) | 2.2 (2.4) | 2.8 (2.4) | 0.216 |

Additional file 1: Table S2 Outcome analysis using proposed propensity score

|  | **No trach** | **Trach** | **p-value** |
| --- | --- | --- | --- |
| Mortality | 29.2% | 14.6% | 0.026 |
| Mortality without comfort care | 3.4% | 4.7% | 0.337 |
| VAP | 14.2% | 37.5% | <0.001 |
| ICU stay, mean days (SD) | 10.0 (6.5) | 27.8 (24.4) | <0.001 |
